# Supplementary material for: The chromatin landscape of healthy and injured cell types in the human kidney
Source: Nat Commun. 2024 Jan 10;15:433. doi: 10.1038/s41467-023-44467-6 (PMC10781985; doi:10.1038/s41467-023-44467-6)
Supplement: Supplementary file 1 — Supplementary Figures [file 41467_2023_44467_MOESM1_ESM.pdf]

Supplementary Information

Supplementary Figures

The chromatin landscape of healthy and injured cell types in the human kidney

Debora L. Gisch[1]*, Michelle Brennan[2]*, Blue B. Lake[3]*, Jeannine Basta[4]*, Mark S. Keller[5], Ricardo Melo Ferreira[1], Shreeram Akilesh[6], Reetika Ghag[4], Charles Lu[4], Ying-Hua Cheng[1], Kimberly S. Collins[1], Samir V. Parikh[7], Brad H. Rovin[7], Lynn Robbins[8], Lisa Stout[4], Kimberly Y. Conklin[3], Dinh Diep[3], Bo Zhang[4], Amanda Knoten[4], Daria Barwinska[1], Mahla Asghari[1], Angela R. Sabo[1], Michael J. Ferkowicz[1], Timothy A Sutton[1], Katherine J Kelly[1], Ian H. De Boer[6], Sylvia E. Rosas[9], Krzysztof Kiryluk[10], Jeffrey B. Hodgin[11], Fadhl Alakwaa[11], Seth Winfree[12], Nichole Jefferson[13], Aydın Türkmen[14], Joseph P. Gaut[3], Nils Gehlenborg[5], Carrie L. Phillips[1], Tarek M. El-Achkar[1], Pierre C. Dagher[1], Takashi Hato[1], Kun Zhang[3], Jonathan Himmelfarb[6], Matthias Kretzler[11], Shamim Mollah[4], the Kidney Precision Medicine Project (KPMP), Sanjay Jain[4]#, Michael Rauchman[4]#, Michael T. Eadon[1]#

*= These authors contributed equally
[1]Indiana University School of Medicine, Indianapolis, IN, 46202, USA
[2]Saint Louis University, St. Louis, MO 63103, USA
[3]Department of Bioengineering, University of California, San Diego, La Jolla, CA, USA
Present address: San Diego Institute of Science, Altos Labs, San Diego, CA, USA
[4]Washington University in Saint Louis, St. Louis, MO 63103, USA
[5]Harvard Medical School, Boston, MA 02142, USA
[6]University of Washington – Seattle, WA 98195, USA
[7]Ohio State University Wexner Medical Center, Columbus, OH 43210, USA
[8]St. Louis Veteran Affairs Medical Center, St. Louis, MO 63106, USA
[9]Joslin Diabetes Center, Harvard Medical School, Boston, MA 02215, USA
[10]Columbia University, New York, NY 10032, USA
[11]University of Michigan, Ann Arbor, MI 48109, USA
[12]University of Nebraska Medical Center, Omaha, NE 68198, USA
[13]Kidney Precision Medicine Project Community Engagement Committee, Dallas TX, USA
[14]Istanbul School of Medicine, Division of Nephrology, Istanbul, Turkey

#= These authors jointly supervised this work

Supplementary Figure 1

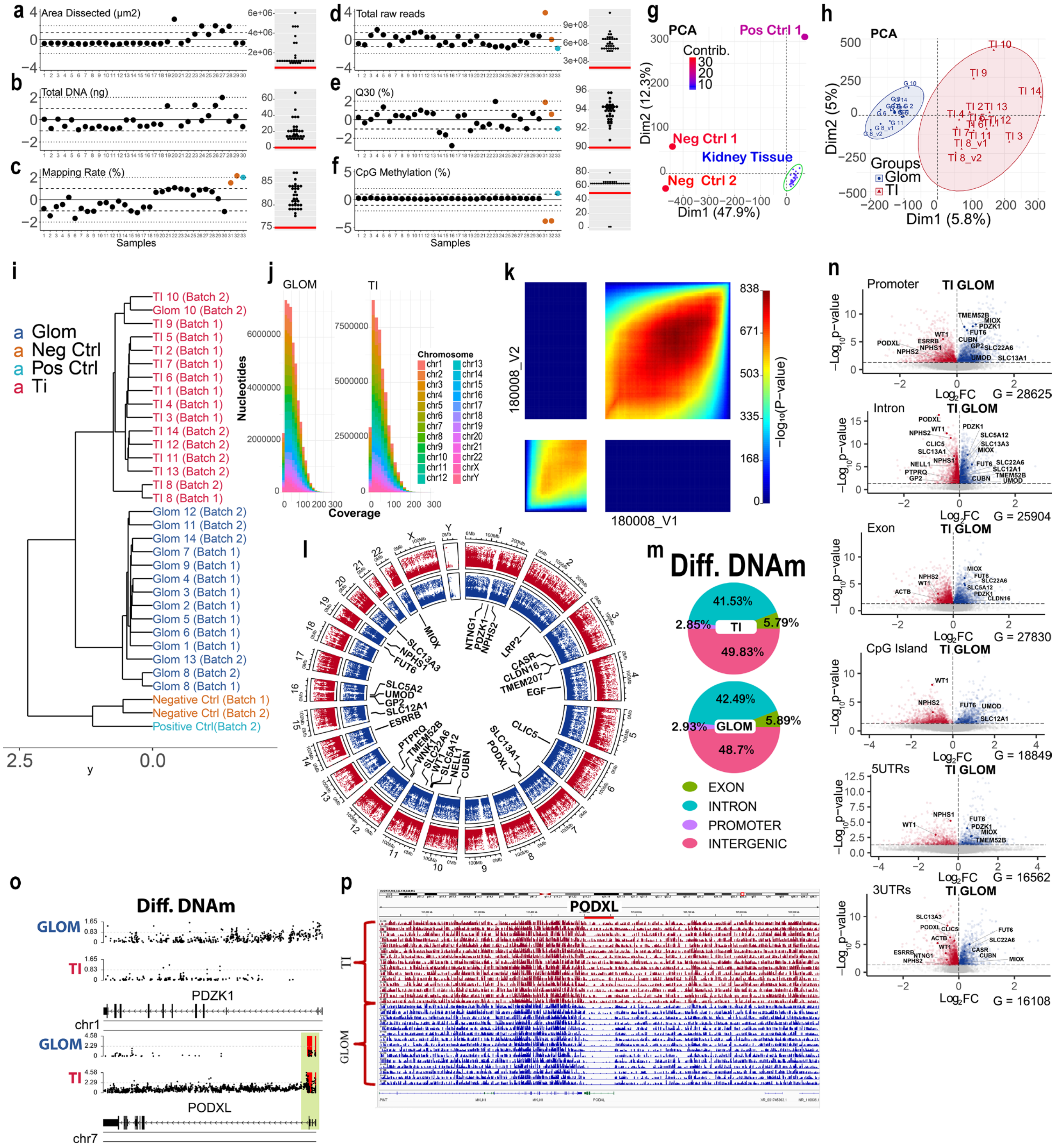

Supplementary Figure 1: Quality control (QC) for whole genome bisulfite sequence (WGBS) in 30 samples, 2 Negative and 1 Positive Control with read coverage > 5. (A-F) The left side shows the mean and standard deviation. The minimum threshold withered line is plotted on the right. Levey-Jennings plots for (**a**) microdissected area: 500,000 μm2, (**b**) DNA input: 0.1 ng, (**c**) Total raw reads in FASTQ: 200M

reads (**d**) percentage of reads with Phred score greater than 30: 90%, (**e**) percentage of mapped reads: 75%, (**f**) CpG Percentage of Methylation > 50%. The positive control is appropriate at ~80% (negative <2%). WGBS samples reproducibly clustered according to biological properties: (**g**) Principal component analyses (PCA) with glomerulus (GLOM), tubulointerstitium (TI), negative and positive controls. (**h**) PCA with glomerulus GLOM and TI (**i**) Hierarchical clustering by methylation levels at nucleotide resolution of GLOM and TI samples using the correlation distance method and ward clustering method. (**j**) Coverage of CpG nucleotides reads showing the distribution along chromosomes. (**k**) Replicate analysis using the Rank Rank Hypergeometric Overlap (RRHO) presents a heatmap with the significance level of overlap between genes of the same sample run in separate batches. (**l**) A circosplot depicts the average methylome of the GLOM and TI across the genome. Differential DNA methylation (DNAm) is plotted for GLOM (blue) and TI (red) P value<0.05 by t-test. (**m**) Percentage of differential DNAm regions by gene annotation. Similar proportions were observed between the GLOM and TI. (**n**) Volcano plots showing differential DNAm for GLOM (blue) and TI (red) in genome regions: promoter, Intron, Exon, CpG island, 5UTRs, 3UTRs P value<0.05 by t-test. (**o**) Differential DNAm in *PDZK1* is increased in GLOM and *PODXL* in TI. (**p**) The gene *PODXL* showed reduced methylation levels in GLOM. 15 GLOM and 15 TI samples, blue and red, respectively, ordered from highest to lowest methylation levels in the *PODXL* gene region show biological reproducibility.

Supplementary Figure 2

a

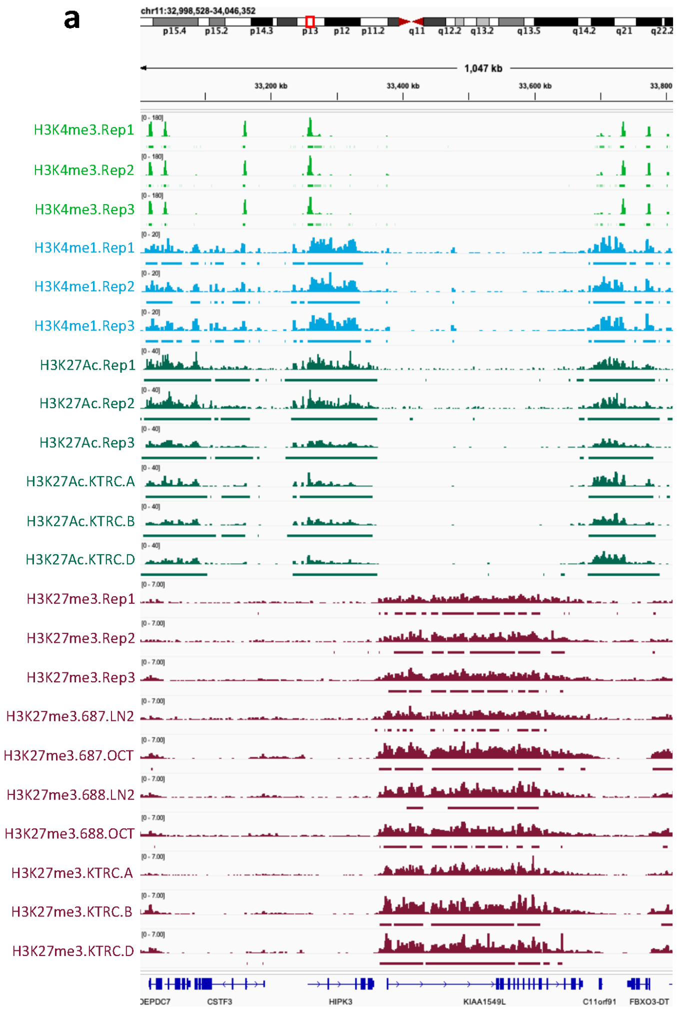

c d e f

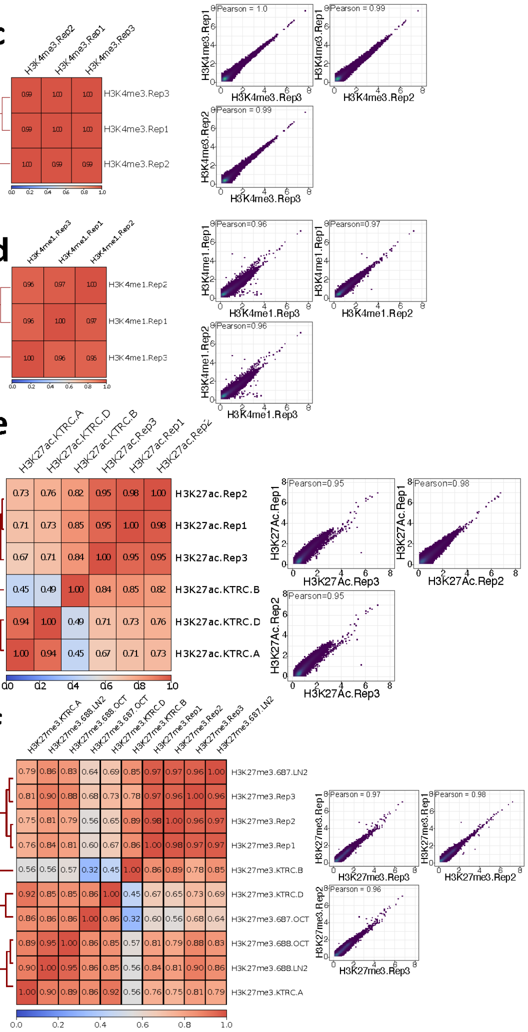

b

Sample	# Cells	Total DNA Recovery (ng)	Total Reads	Percent Aligned	Average Insert Size	Percent Reads in Peaks
H3K4me3.Rep1	150,000	3.42	37,399,954	91.7	172	61.0
H3K4me3.Rep2	150,000	2.24	26,592,280	89.8	171	65.2
H3K4me3.Rep3	150,000	2.85	26,893,215	89.8	172	59.7
H3K4me1.Rep1	150,000	1.1	48,737,316	97	174	55.1
H3K4me1.Rep2	150,000	out of range	44,648,476	96.9	170	56.4
H3K4me1.Rep3	150,000	7.44	26,780,397	95.2	172	63.8
H3K27Ac.Rep1	150,000	2.18	41,269,234	90.7	163	68.6
H3K27Ac.Rep2	150,000	2.46	35,350,160	93.5	168	67.8
H3K27Ac.Rep3	150,000	out of range	47,437,491	96.2	170	46.1
H3K27Ac.KTRC.A	150,000	1.37	123,727,054	93.5	165	73.7
H3K27Ac.KTRC.B	150,000	1.86	124,954,822	92.8	166	64.2
H3K27Ac.KTRC.D	150,000	3.64	127,335,411	94.1	179	70
H3K27me3.Rep1	100,000	2.92	42,970,021	95	170	70.3
H3K27me3.Rep2	100,000	3.02	44,290,646	96.4	171	69.4
H3K27me3.Rep3	150,000	8.52	39,747,143	98.5	176	55
H3K27me3.687.LN2	50,000	8.2	24,815,292	98.6	175	55.4
H3K27me3.687.OCT	200,000	49.6	43,963,321	98	163	44.5
H3K27me3.688.LN2	66,000	8.8	58,459,237	98.7	172	50.5
H3K27me3.688.OCT	66,000	2.64	40,181,951	97.7	167	50.4
H3K27me3.KTRC.A	150,000	2.54	147,088,937	94.9	168	22.8
H3K27me3.KTRC.B	150,000	3.74	104,349,021	94.6	167	44.4
H3K27me3.KTRC.D	150,000	9.16	118,563,576	95.4	183	38.5

g

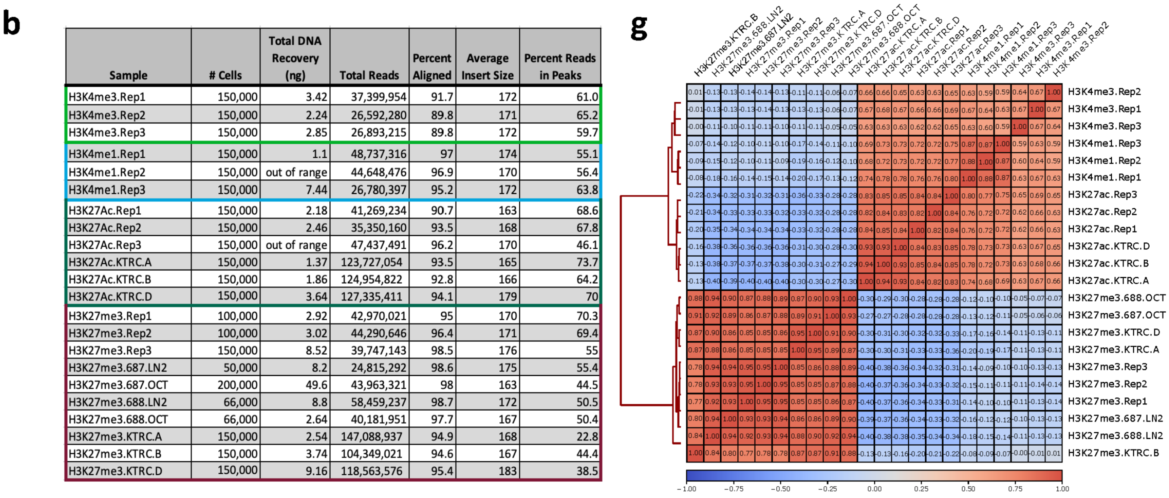

Supplementary Figure 2: Quality control measures for CUT&RUN samples. (**a**) Representative tracks of a genomic region in chromosome 11 for H3K4me3 (3 technical replicates), H3K4me1 (3 technical replicates), H3K27ac (4 biological replicates and 3 technical replicates), and H3K27me3 (6 biological replicates and 3 technical replicates). Within the genomic region is a gene expressed within the kidney (*HIPK3*) that has H3K4me3 at the promoter, with H3K4me1 downstream of the promoter and within the gene body, and H3K27ac at the promoter and gene body. In contrast, adjacent to *HIPK3* is the gene *KIAA1549L*, which has very low/no expression in the kidney. This gene is devoid of H3K4me3, H3K4me1, and H3K27ac, but is enriched for H3K27me3. MACS2 called peaks for each track are below the CUT&RUN track for each sample. (**b**) Table with CUT&RUN samples used in this study, including the number of cells used for each CUT&RUN reaction, DNA recovered in the CUT&RUN reaction (ng), total reads for each library, % aligned reads, average insert size, and percent reads in peaks. (**c**) Genome wide Pearson correlation for technical replicates of H3K4me3 and (**d**) H3K4me1, biological and technical replicates of (**e**) H3K27ac and (**f**) H3K27me3. (**g**) Genome wide Spearman correlation for all CUT&RUN samples. As expected, H3K27me3 has low correlation with histone modifications associated with active chromatin (H3K27ac, H3K4me3, H3K4me1).

Supplementary Figure 3

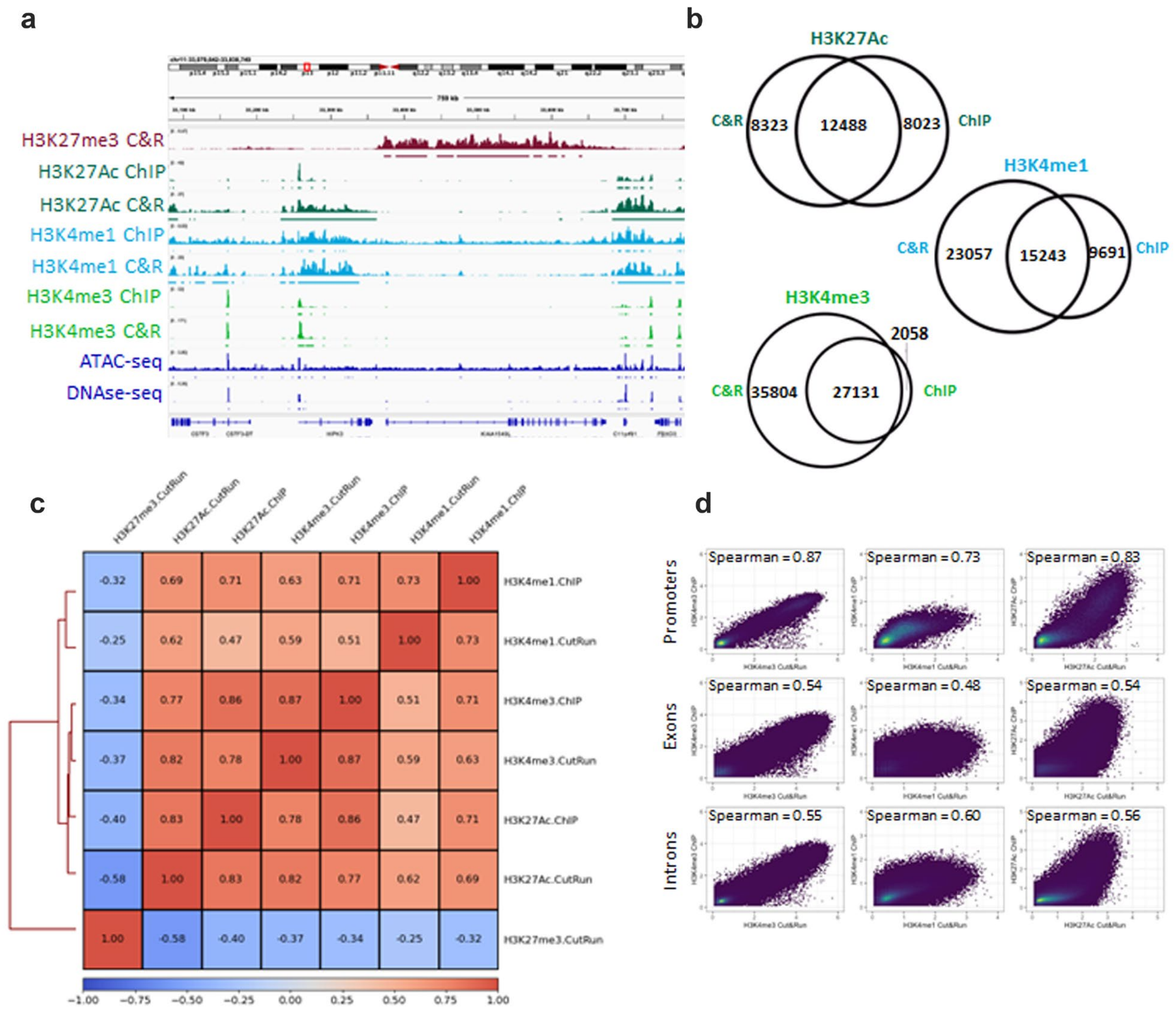

Supplementary Figure 3: CUT&RUN (C&R) validation with ENCODE ChIP-seq. (**a**) Representative tracks of CUT&RUN with a correlating track of ENCODE ChIP-seq (excluding H3K27me3 for which there is no ChIP-seq for adult human kidney in ENCODE). ENCODE H3K27ac- ENCFF354FYC/ENCFF266MBV, H3K4me1- ENCFF992BPB/ENCFF845DVB, H3K4me3- ENCFF929KNV/ENCFF349XHZ, ATAC-seq- ENCFF395HGS, DNAse-seq- ENCFF485CJZ/ENCFF763YHH. Called peaks are below each CUT&RUN or ChIP-seq track. For each histone modification CUT&RUN reaction there are similar binding patterns compared to the ChIP-seq, although the CUT&RUN reactions have much higher signal with lower background. (**b**) Venn diagrams comparing called peaks for H3K27ac, H3K4me1, and H3K4me3 from CUT&RUN and ChIP-seq. (**c**) Genome wide Spearman correlation for CUT&RUN and ENCODE ChIP-seq. As expected, H3K27me3 has a negative correlation with H3K27ac, H3K4me3, and H3K4me1 histone modifications. (**d**) Spearman correlation for H3K4me3, H3K4me1, and H3K27ac at promoters, exons, and introns.

Supplementary Figure 4

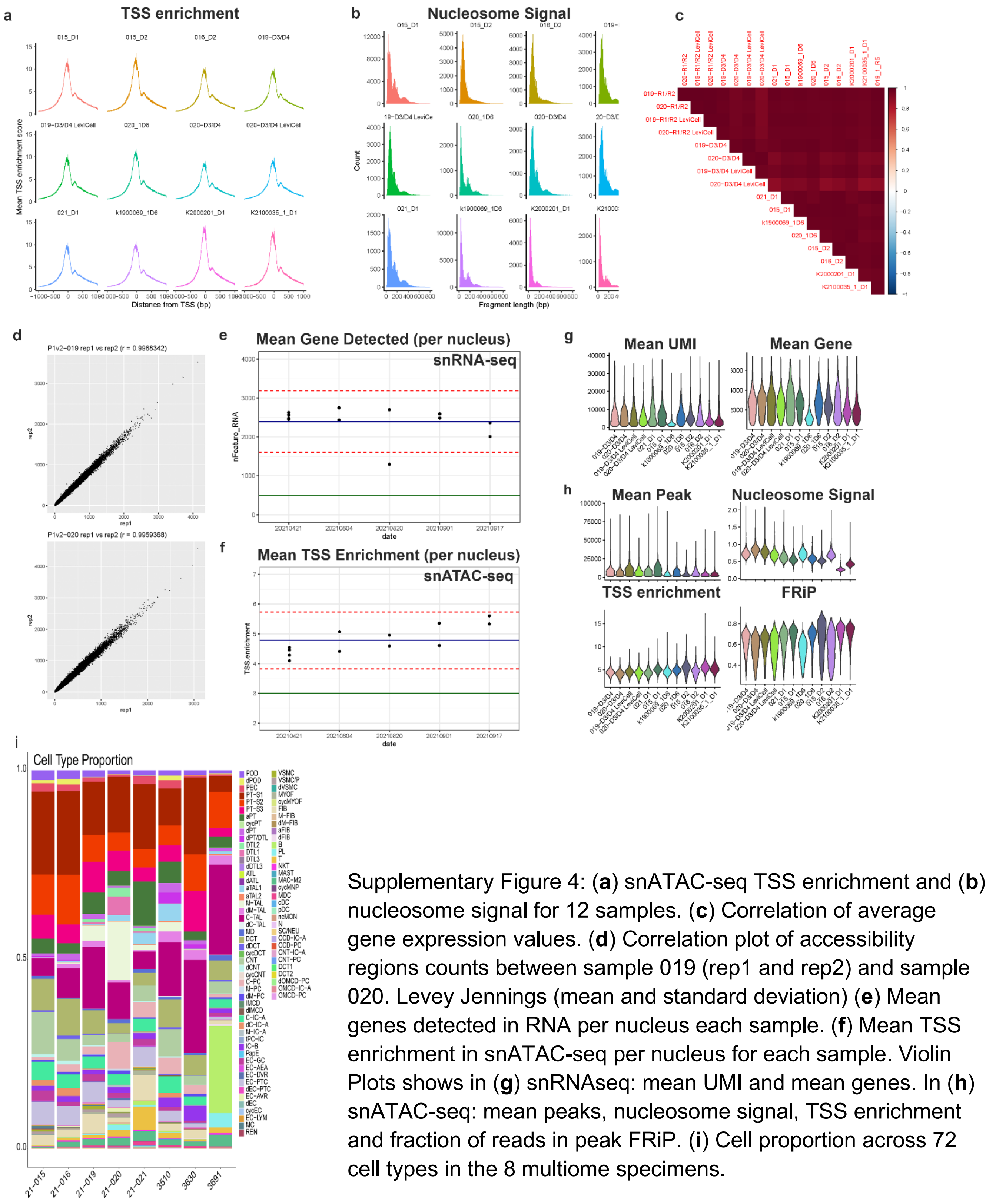

Supplementary Figure 4: (**a**) snATAC-seq TSS enrichment and (**b**) nucleosome signal for 12 samples. (**c**) Correlation of average gene expression values. (**d**) Correlation plot of accessibility regions counts between sample 019 (rep1 and rep2) and sample 020. Levey Jennings (mean and standard deviation) (**e**) Mean genes detected in RNA per nucleus each sample. (**f**) Mean TSS enrichment in snATAC-seq per nucleus for each sample. Violin Plots shows in (**g**) snRNAseq: mean UMI and mean genes. In (**h**) snATAC-seq: mean peaks, nucleosome signal, TSS enrichment and fraction of reads in peak FRiP. (**i**) Cell proportion across 72 cell types in the 8 multiome specimens.

Supplementary Figure 5

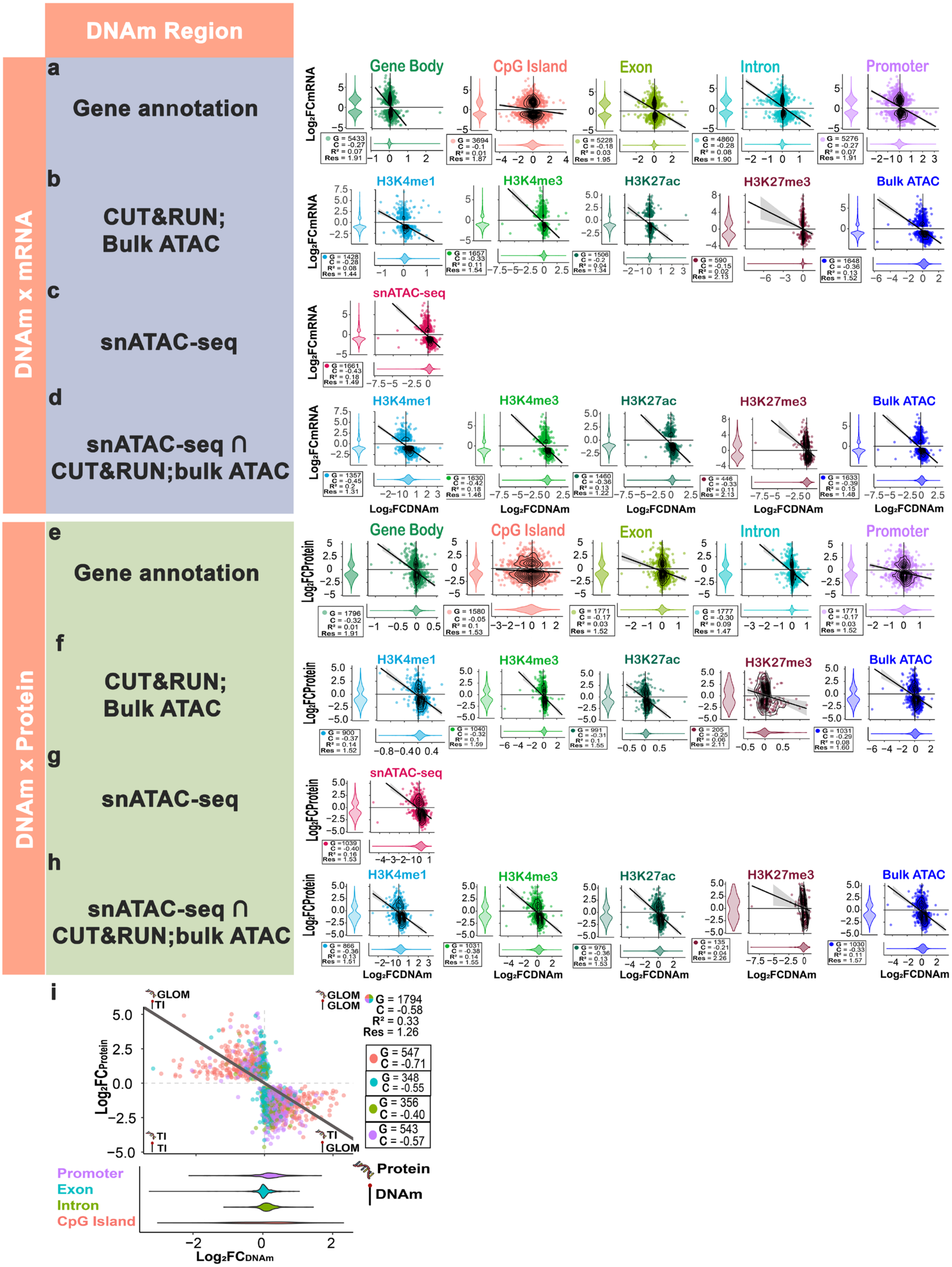

Supplementary Figure 5: (DNAm (N=30) + CUT&RUN (N=22) + snATAC-seq (N=12)) Linear regression between Differential DNAm and mRNA (N=22) or protein (N=12) for two compartments GLOM (+) and TI (-), with the number of elements with each annotation, correlation, R squared and residuals. (**a**)(**e**) by gene annotation: gene body; CpG Island; exon; Intron; promoter. (**b**)(**f**) Histone mark peaks for H3K4me1, H3K4me3, H3K27ac, H3K27me3, respectively and bulk ATAC peaks. (**c**)(**g**) multiome snATAC-seq open chromatin and (**d**)(**h**) regions with signal overlapping between multiome open chromatin peaks and histone modification peaks. (**i**) The optimal relationship between differential methylation and protein occurred in regions G=547 CpG Island, G=348 exon, G= 356 intron, G=547 promoter, generating correlations by gene annotation of the C=-0.71, C=-0.55, C=-0.40, and C=-0.57 respectively. The correlation between differential DNAm and protein with G=1794 elements using the optimal region of each gene results in a correlation of C=-0.58, R square R2=0.33, and Residual Res=1.26. The figure uses a stock image from Adobe Illustrator with license (Eadon et. al – stock.adobe.com).

Supplementary Figure 6

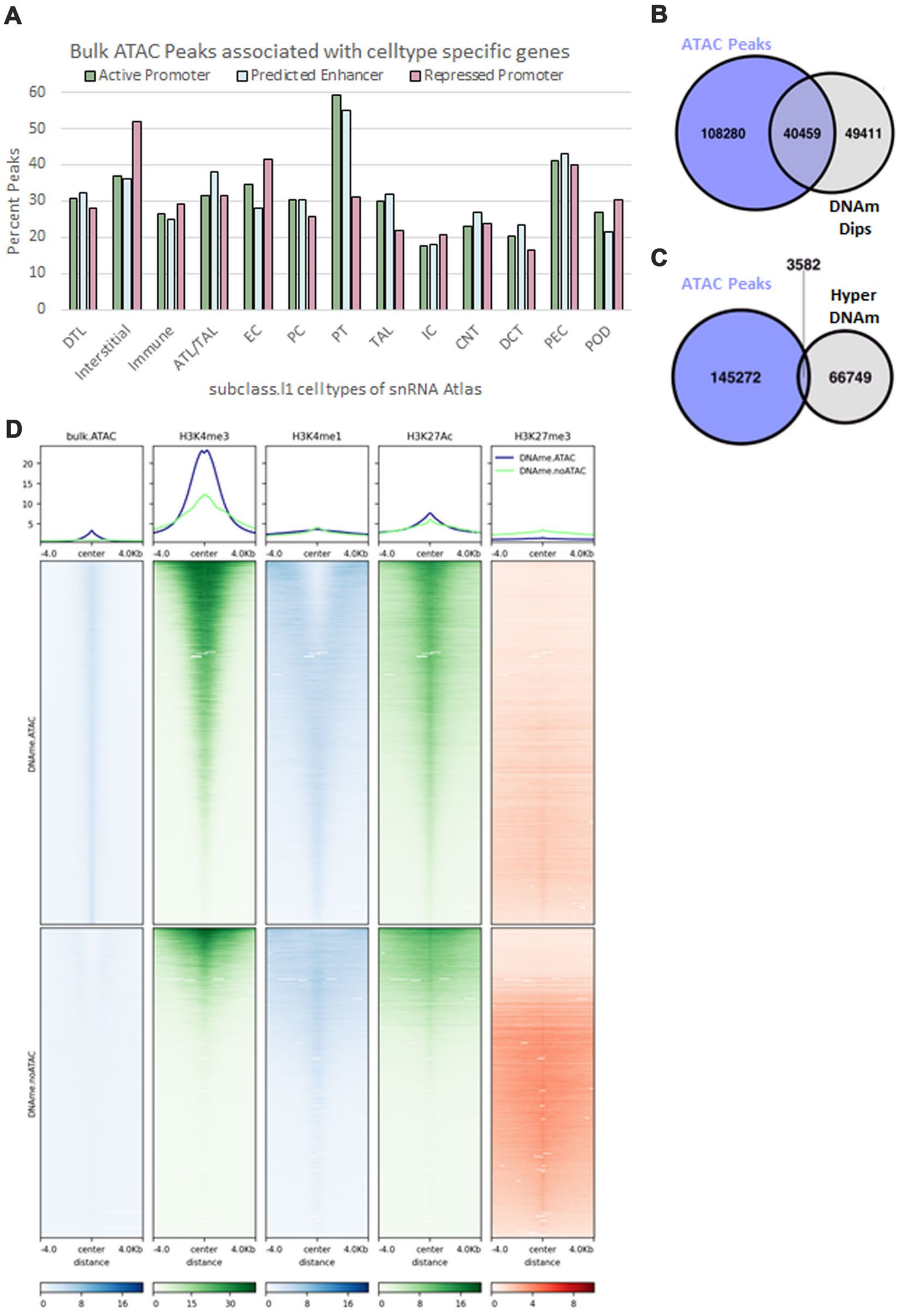

Supplementary Figure 6: (**a**) Distribution of bulk ATAC-seq peaks (N=1) for promoters and enhancers in cell specific marker genes. The distribution reflects the cell type abundance in whole kidney. (**b-c**) Venn diagrams comparing bulk ENCODE (ENCFF395HGS) ATAC peaks with reduced DNA methylation (dips, DNAme value of <= 0.4 across 50bp) or elevated DNA methylation (DNAme value of >= 0.8 across 50bp) regions. (**d**) Heatmap of CUT&RUN (N=22) histone marks in DNA methylation TI dips (N=15) including (top) or excluding (bottom) an ATAC-seq open chromatin peak.

Supplementary Figure 7

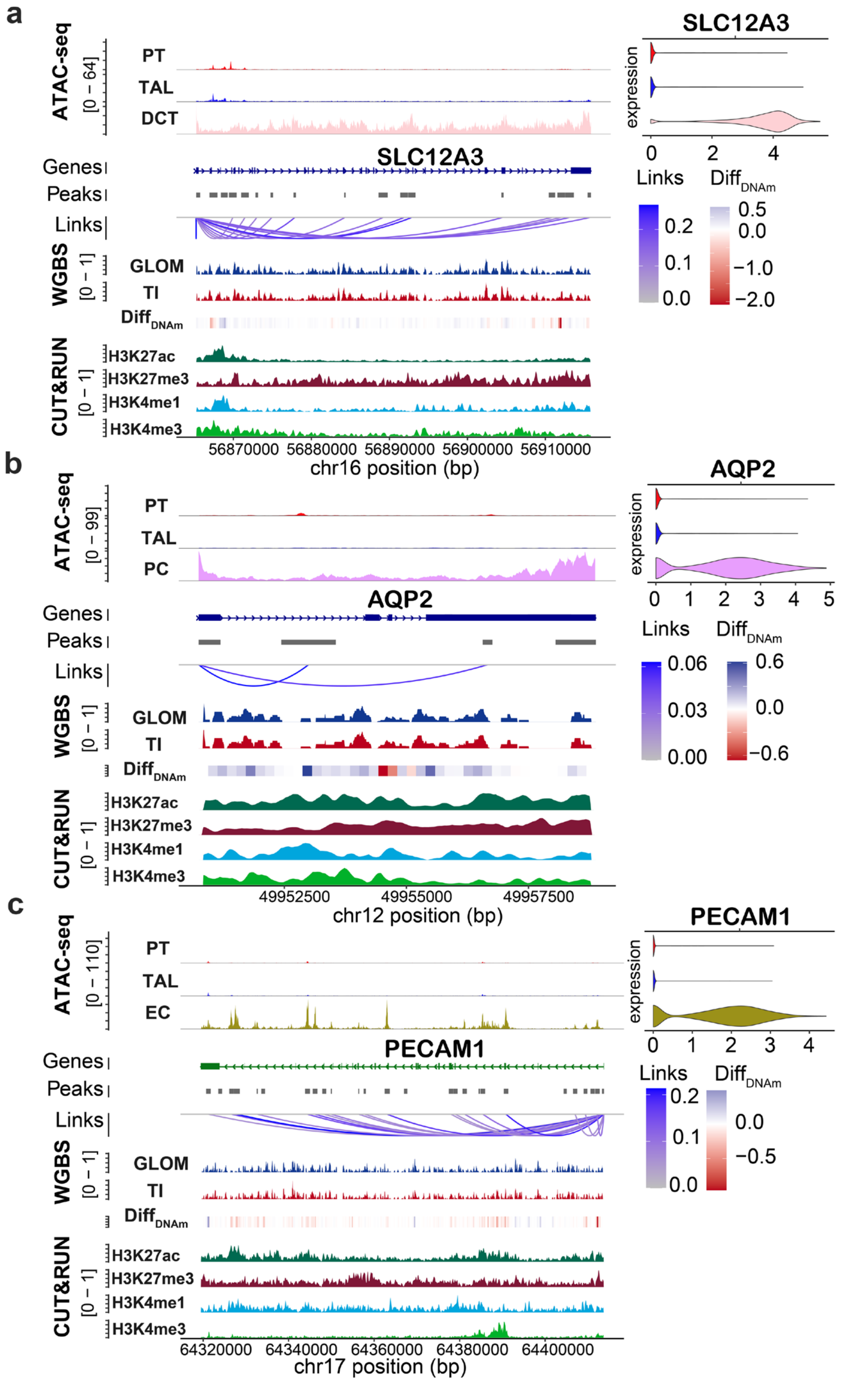

Supplementary Figure 7: Alignment of epigenomic features across three marker genes: (**a**) *SLC12A3* in Distal Convolution Tubule (DCT) (**b**) *AQP2* in principal cells (PC) (**c**) *PECAM1* epithelium cells (EC) compared to the proximal tubule (PT) and thick ascending loop of Henle (TAL). Tracks are seen for multiome (N=12)open chromatin signal, gene expression (N=12), DNA methylation (N=30), and CUT&RUN (N=22).

Supplementary Figure 8

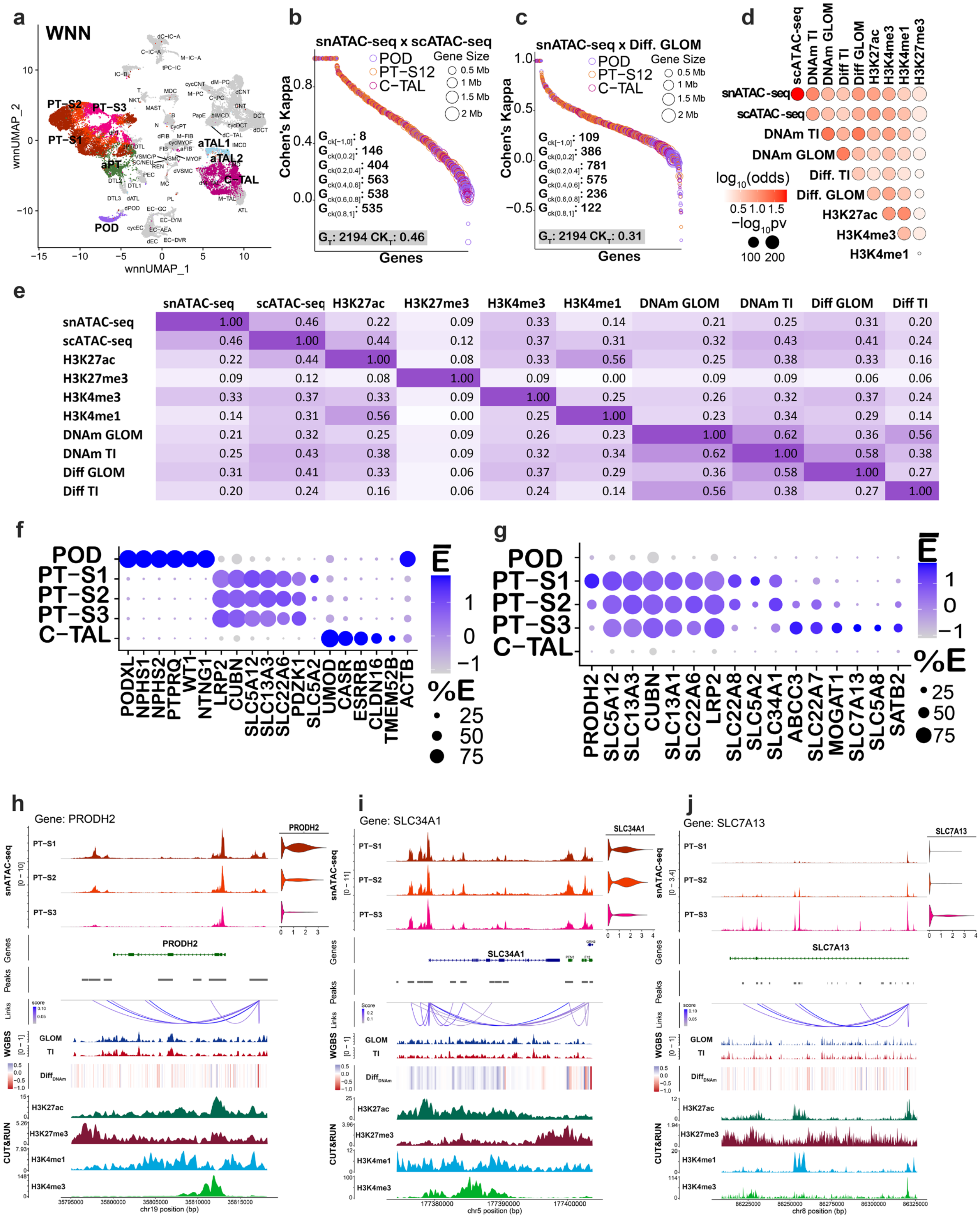

Supplementary Figure 8: (**a**) UMAP WNN reduction for 72 cell types (N=12). Cohen's Kappa for (**b**) multiome snATAC-seq compared to publicly available scATAC-seq (Humphrey's lab, N=5). (**c**) multiome snATAC-seq and differential DNA methylation (DNAm) in the glomerulus (GLOM) (N=15). (**d**) Fischer exact test for all technologies aligned across hg38 corresponding to Figure 3I but adding Diff. DNAm (GLOM and TI) (N=30) and snATAC-seq[5] (GSE151302) merged with snRNA[3] to validate the multiome snATAC-seq. (**e**) The matrix shows the global Cohen's Kappa combining all results where the technologies are analyzed two by two. The Cohen's Kappa range goes between total agreement (1) and total disagreement (-1). (**f**) Average and percent mRNA expression of the same genes from Figure 3c in POD, PT-S1, PT-S2, PT-S3, and C-TAL. (**g)** Dotplot for PT marker genes. (**h-j**) Alignment of epigenomic technologies, snATAC (N=12), DNAm (N=30) and histone marks (N=22), for gene markers of PT (S1, S2 and S3) *PRODH2*, *SLC34A1* and *SLC7A13*. Heat map presents Diff DNAm GLOM/TI with positive values indicating a higher methylation in GLOM.

Supplementary Figure 9

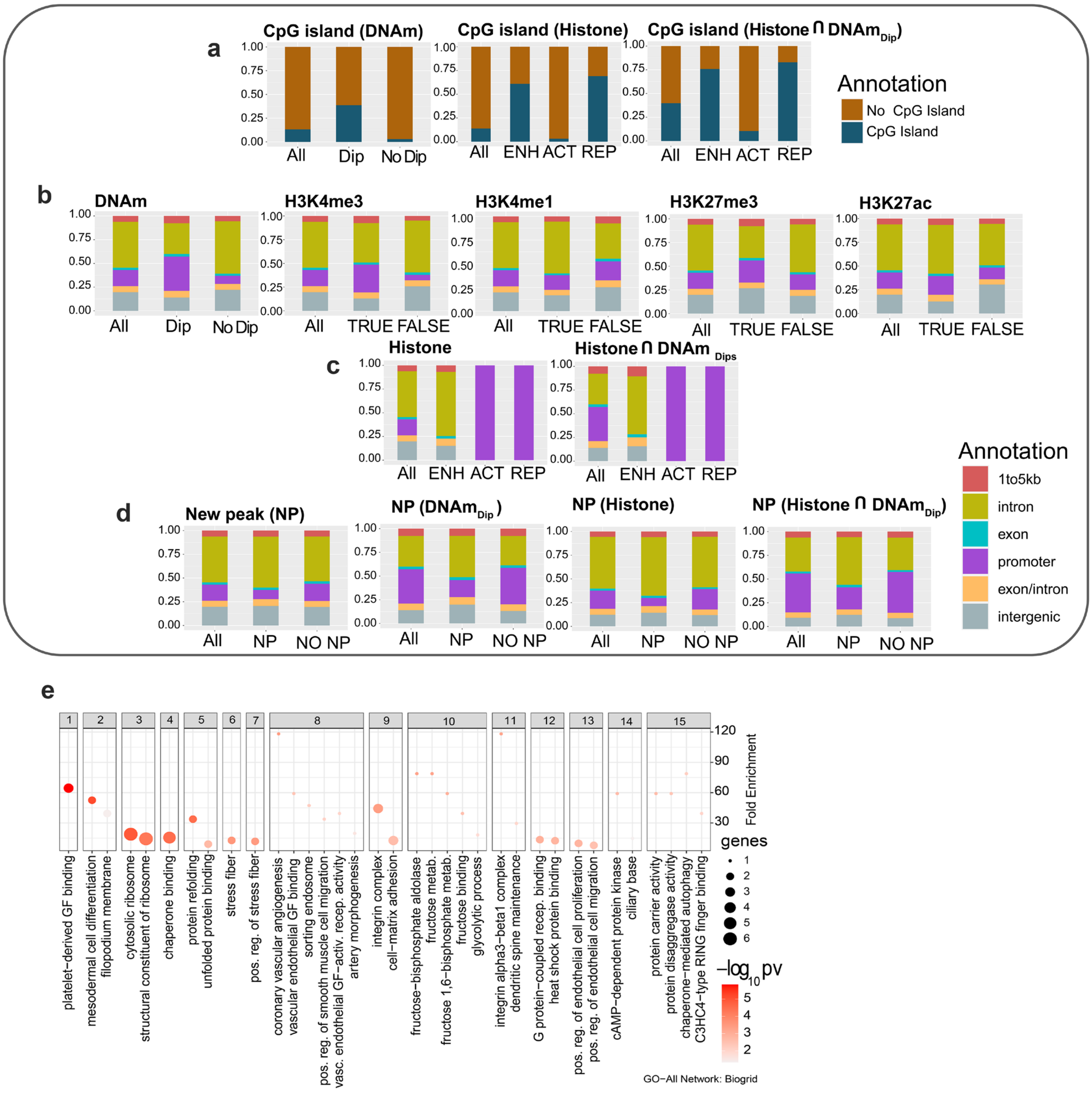

Supplementary Figure 9: Gene annotation proportion for adaptive proximal tubule (aPT) and PT peaks from multiome altlas (N=12). (**a**) The CpG island annotation of peaks across: DNAmTI (N=15) (Dips and No Dips), peaks with histone mark interpretation and peaks with simultaneous Dips in DNAm and interpretation of histone marks. (**b**) DNAm TI (Dips and No Dips), histone marks H3K4me3 (N=3), H3K4me1 (N=3), H3K27me3 (N=6) and H3K27ac (N=10) (All peaks, peaks with signal TRUE for present and FALSE for absent). (**c**) Peaks with an interpretation across the histone mark intersections. Active promoter = peak annotation is within a

promoter region and positive for H3K4me3, H3K4me1, and H3K27ac. Repressed promoter= peak annotation is within a promoter region, negative for H3K27ac, and positive for H3K27me3. Enhancer= peak annotated outside of a promoter region positive for both H3K4me1 and H3K27ac. (**d**) Peaks with interpretation and DNAm TI dips across the histone mark intersections. (**e**) Top 15 clusters from GO-All pathway enrichment analysis based on DA. Key pathways of the adaptive process include platelet-derived growth factor binding, mesodermal cell differentiation, and integrin complex and adhesion.

Supplementary Figure 10

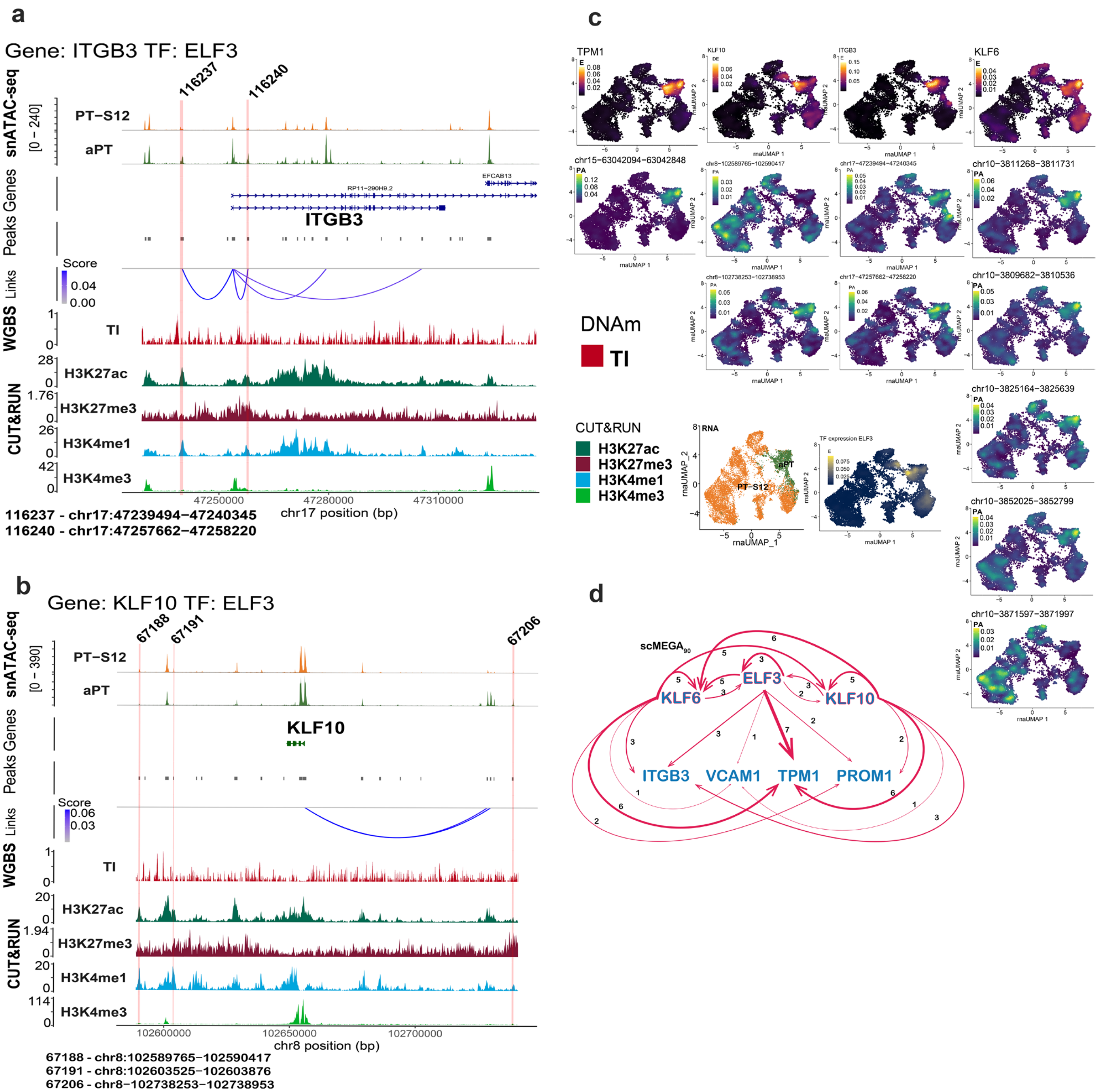

Supplementary Figure 10: (**a-b**) Alignment of epigenomic features from multiome atlas (N=12) in *ITGB3* and *KLF10* for the aPT and PT-S12. Red stripe indicates a peak with predicted transcription factor (TF) binding by *ELF3*. TF Peaks are numbered and correspond to (Supplementary Table 6). (**c**) UMAP RNA with expression, accessibility, and binding by *ELF3* for *TPM1*, *KLF10*, *ITGB3* and *KLF6. ITGB3* peaks and predicted binding by *ELF3*, accessibility of *KLF10* peaks with predicted binding by *ELF3*. (**d**) Gene regulatory network predicted by scMEGA for the TF *KLF10*, *KLF6* and *ELF3*.

Supplementary Figure 11

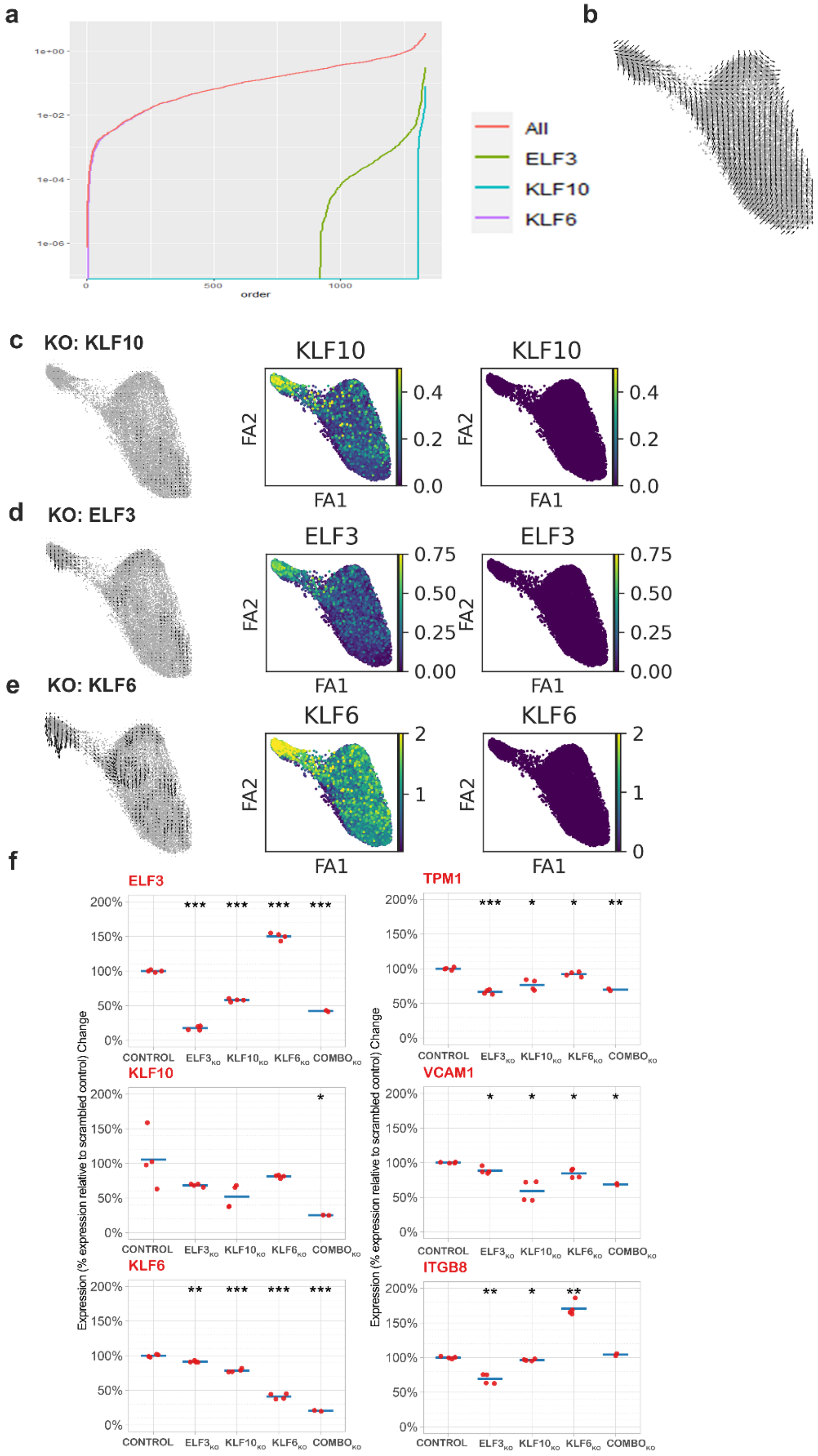

Supplementary Figure 11: Individual *in silico* perturbation knockouts in the proximal tubule (PT) cells from multiome atlas (N=12). (**a**) Contribution of each gene knockout to changes in the adaptive proximal tubule (aPT) gene expression signature. *KLF6* was predicted to affect the greatest quantity of aPT genes, but *ELF3* was also predicted to have unique effects on the aPT gene signature. The contribution of *KLF10* was minimal. (**b**) Cell flow trajectory before knockout. (**c-e**) Trajectory change after isolated knockout of (**c**) *ELF3*, (**d**) *KLF6*, and (**e**) *KLF10* with expression before and after individual knockout. (**f**) siRNA knockdown in normal human proximal tubule kidney cells. Knockdown of ELF3, KLF10, KLF6, and their combination reveal a complex regulatory relationship of each other and the aPT target genes *ITGB8*, *TPM1*, and *VCAM1*. The combined knockdown led to reduction of *TPM1* and *VCAM1* expression. *ELF3* knockdown led to reduction of *ITGB8* and *TPM1* expression, while *KLF10* knockdown led to reduced *VCAM1* expression. *KLF6* knockdown did not reduce aPT target gene expression. We performed t-tets to obtain the significance levels for the P values. They are denoted as follows: P value <0.05 is represented by '*', *P value <0.005 by* '**', ***and*** *P value<0.0005* **by** '***'. Each gene has Control (N=4), ELF3 ko (N=4), KLF10 ko (N=4), KLF6 ko (N=4) and COMBO (N=2).

Supplementary Figure 12:

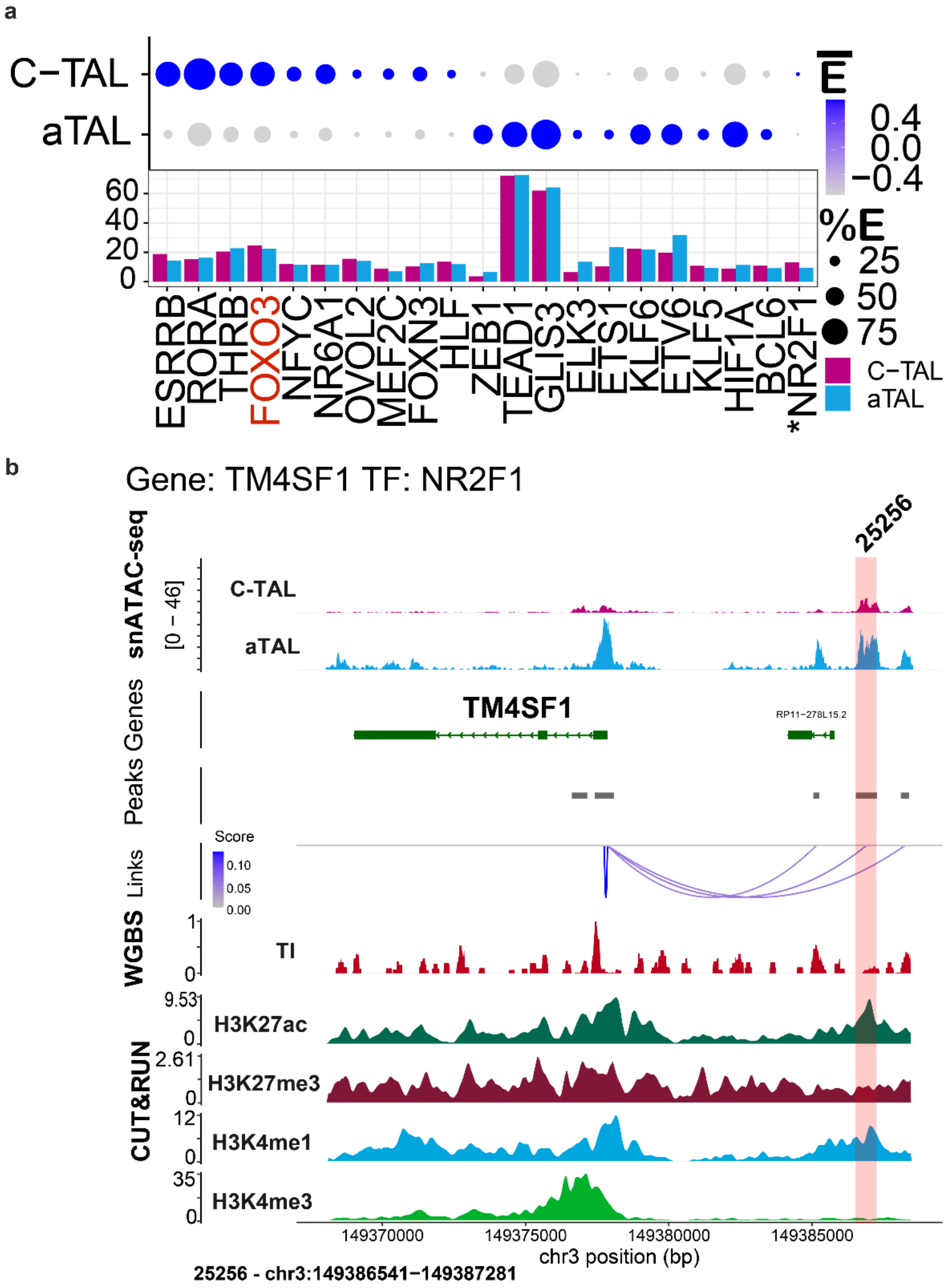

Supplementary Figure 12: (**a**) Dotplot of the 10 most differential expressed TF in snRNA-seq (N=12) for aTAL and C-TAL cell type in scMEGA analyse. *NR2F1* TF is not differential expressed. Bar plot showing area under the curve (AUC) of average counts. *FOXO3* (red) is highly expressed in scMEGA, but not identified amongst TRIPOD results. (**b**) Alignment of epigenomic feature in *TM4SF1* for the aTAL and C-TAL.
