# Supplementary material for: The chromatin landscape of healthy and injured cell types in the human kidney
Source: Nat Commun. 2024 Jan 10;15:433. doi: 10.1038/s41467-023-44467-6 (PMC10781985; doi:10.1038/s41467-023-44467-6)
Supplement: Supplementary file 3 — Description of Additional Supplementary Files [file 41467_2023_44467_MOESM3_ESM.pdf]

Description of Additional Supplementary Files

Supplementary Data 1: Clinical data

Description: The clinical data presented in the table provides a concise summary of important patient information relevant to the study. This data includes key demographic details, medical history, and clinical observations.

Supplementary Data 2: Differential Methylation between GLOM/TI

Description: This table provides a comprehensive overview of differential methylation analysis between Glomerular (GLOM) and Tubulointerstitial (TI) regions in the context of genomic features. Methylation levels were calculated and aggregated within distinct genomic regions in the hg38 reference genome.

Supplementary Data 3: Kappa Cohen's results

Description: Cohen's Kappa values, which measure the degree of agreement or not, for pairwise comparisons of 2224 genes expressed in (PT-S1S2, C-TAL or POD) across various technologies.

Supplementary Data 4: Differential expressed gene aPT x PTS1S2 (DE)

Description: the results of the analysis of the genes between the adaptive/maladaptive PT and the reference PTS1S2 (DE) using two different statistical tests, the Wilcox Test with Bonferroni correction (adjusted p value <0.05) and the Negative Binomial Test with Bonferroni correction (adj.pv<0.05), with an additional criterion of absolute average log2 fold change (avg log2FC) greater than 0.25.

Supplementary Data 5: Differential accessibility (DA) peaks between aPT x PT-S1S2

Description: Differential Accessibility (DA) Peaks observed between the adaptive Proximal Tubule (aPTx) and the reference PT-S1S2 (PT-S1S2) conditions, providing detailed information on the genomic locations and characteristics of DA peaks, including their statistical significance.

Supplementary Data 6: Summarizing TRIPOD results for aPTxPT-S1S2

Description: The TRIPOD table allows researchers to assess the statistical significance of integrated findings from the TRIPOD analysis, including differential accessibility peaks, differential expression genes, and the binding of genes by transcription factors in the context of the aPTxPT-S1S2 comparison.

Supplementary Data 7: Enrichment analysis results from pathfindR

Description: Results of the enrichment analyses for gene ontology in GO-ALL conducted using pathfindR v.2.1.0 for genes upregulated in aPT clusters with transcription factor (TF) binding in

regions of differential accessibility. The p-values obtained from the enrichment tests have been adjusted using the Bonferroni method for multiple testing correction.

Supplementary Data 8: Summarizing scMEGA results for aPT x PT-S1S2

Description: scMEGA analysis, including correlations between genes and transcription factors, and their significance in the context of the aPTxPT-S1S2 comparison. It also highlights genes targeted by transcription factors.

Supplementary Data 9: TF Activity Matrix for aPTxPT-S1S2

Description: TF (Transcription Factor) Activity Matrix constructed by scMEGA for the aPTxPT-S1S2 comparison. The matrix has been filtered to retain only interactions between TFs and target genes with a correlation of greater than 90%.

Supplementary Data 10: Differential Expressed Gene before and after In Silico Knockout (KO)

Description: Provides information about differential expression analysis for genes before and after an in silico knockout (KO) in KLF6, KLF10, ELF3 or (KLF6_KLF10_ELF3)

Supplementary Data 11: Differential expressed gene aTAL x C-TAL (DE)

Description: Results of the analysis of the gene differential expression (DE) of the adaptive thick ascending limb of the loop of Henle (aTAL) and the healthy reference cortical TAL (C-TAL) using a Wilcox Test with Bonferroni correction (adjusted p value <0.05).

Supplementary Data 12: Enrichment Analysis Results for Upregulated Genes in aTAL

Description: Presents the results of enrichment analyses conducted using pathfindR for genes that are upregulated in the adaptive thick ascending limb of the loop of Henle (aTAL). The p-values obtained from the enrichment tests have been adjusted using the Bonferroni method for multiple testing correction.

Supplementary Data 13: Summarizing TRIPOD results for aTAL x C-TAL

Description: The TRIPOD table allows the assessment of the statistical significance of the integrated findings from TRIPOD analysis, including differential accessibility peaks, differential expression genes, and the binding of genes by transcription factors in the context of aTALxC-TAL comparison.

Supplementary Data 14: Summarizing scMEGA results for aTAL x C-TAL

Description: An scMEGA analysis, including correlations between genes and transcription factors, and their significance in the context of the aTALxC-TAL comparison. It also highlights genes targeted by transcription factors.

Supplementary Data 15: TF Activity Matrix for aTAL x C-TAL

Description: TF (Transcription Factor) Activity Matrix constructed by scMEGA for the aTAL x C-TAL comparison. The matrix has been filtered to retain only interactions between TFs and target genes with a correlation of greater than 90%.
