# Supplementary material for: The chromatin landscape of healthy and injured cell types in the human kidney
Source: Nat Commun. 2024 Jan 10;15:433. doi: 10.1038/s41467-023-44467-6 (PMC10781985; doi:10.1038/s41467-023-44467-6)
Supplement: Supplementary file 6 — Reporting Summary [file 41467_2023_44467_MOESM6_ESM.pdf]

nature portfolio

Corresponding author(s): Michael Eadon, Michael Rauchman, Sanjay Jain

Last updated by author(s): Nov 29, 2023

Reporting Summary

Nature Portfolio wishes to improve the reproducibility of the work that we publish. This form provides structure for consistency and transparency in reporting. For further information on Nature Portfolio policies, see our Editorial Policies and the Editorial Policy Checklist.

Statistics

For all statistical analyses, confirm that the following items are present in the figure legend, table legend, main text, or Methods section.

n/a	Confirmed	
☐	☒	The exact sample size (*n*) for each experimental group/condition, given as a discrete number and unit of measurement
☐	☒	A statement on whether measurements were taken from distinct samples or whether the same sample was measured repeatedly
☐	☒	The statistical test(s) used AND whether they are one- or two-sided<br>*Only common tests should be described solely by name; describe more complex techniques in the Methods section.*
☒	☐	A description of all covariates tested
☐	☒	A description of any assumptions or corrections, such as tests of normality and adjustment for multiple comparisons
☐	☒	A full description of the statistical parameters including central tendency (e.g. means) or other basic estimates (e.g. regression coefficient) AND variation (e.g. standard deviation) or associated estimates of uncertainty (e.g. confidence intervals)
☐	☒	For null hypothesis testing, the test statistic (e.g. *F*, *t*, *r*) with confidence intervals, effect sizes, degrees of freedom and *P* value noted<br>*Give P values as exact values whenever suitable.*
☒	☐	For Bayesian analysis, information on the choice of priors and Markov chain Monte Carlo settings
☐	☒	For hierarchical and complex designs, identification of the appropriate level for tests and full reporting of outcomes
☐	☒	Estimates of effect sizes (e.g. Cohen's *d*, Pearson's *r*), indicating how they were calculated

Our web collection on statistics for biologists contains articles on many of the points above.

Software and code

Policy information about availability of computer code

Data collection	No software was used for data collection
Data analysis	Code is available at https://github.iu.edu/dgisch/gisch-et-al-2023<br><br>This manuscript is supported by a suite of packages that facilitate statistical analyses. In R (v.4.1.0) programming language: Seurat (v.4.0.0) and Signac (v.1.6.0) enable the visualization, interpretation, and integration of Multiome data (snRNA+snATAC) at the single-cell level, bridging the gap between two domains: RNA and chromatin. Annotatr (v.1.21.0) provides consistent gene annotation across all datasets, while pathfindR (v.2.1.0) performs enrichment pathway analyses. Granulator (v.1.7.0) is a tool used for cell deconvolution in bulk ATAC. In Python (v.3.11.4) programming language, MACS (v.3.0.0a6) identifies peaks and dips across the genome for snATAC, DNAm TI and CUT&RUN and CellOracle (v.0.10.12) simulates the in-silico perturbation.

For manuscripts utilizing custom algorithms or software that are central to the research but not yet described in published literature, software must be made available to editors and reviewers. We strongly encourage code deposition in a community repository (e.g. GitHub). See the Nature Portfolio guidelines for submitting code & software for further information.

Data

Policy information about availability of data

All manuscripts must include a data availability statement. This statement should provide the following information, where applicable:

- Accession codes, unique identifiers, or web links for publicly available datasets
- A description of any restrictions on data availability
- For clinical datasets or third party data, please ensure that the statement adheres to our policy

All sequencing data were analyzed using the GRCh38 (hg38) reference genome. BigWig tracks are available for all datasets in the Kidney chromatin landscape browser at https://doi.org/10.48698/HHE6-YV15. Due to privacy considerations and the nature of DNA information, raw FASTQand BAM files are available upon request at www.kpmp.org. Downloadable BigWig files for whole genome bisulfite sequencing and CUT&RUN are available at https://doi.org/10.48698/HHE6-YV15. The expression matrices for the multiome are available at https://doi.org/10.48698/HHE6-YV15. The Seurat object is available in Zenodo at https://doi.org/10.5281/zenodo.8029990. All differentially expressed genes and differentially accessible peaks for all analyses are included in the supplementary tables provided with this paper. Source data files are provided with underlying data for statistics included in figure panels.

Research involving human participants, their data, or biological material

Policy information about studies with human participants or human data. See also policy information about sex, gender (identity/presentation), and sexual orientation and race, ethnicity and racism.

Reporting on sex and gender	Both sexes were included in this study since kidney disease affects males and females. Sex was self identified. The sex of the participants is detailed in Supplemental Table 1.
Reporting on race, ethnicity, or other socially relevant groupings	We reported race when this data was available. Many of the specimens were deidentified, so race and other socially relevant groupings were not available.
Population characteristics	This study focused on reference nephrectomy samples and some diseased kidney biopsy samples. The age of the participants in the tissue studies is provided in Supplemental table 1 as ranges. The histopathologic characteristics of participants in the are provided in Supplemental table 1
Recruitment	Described in methods. Biopsies were during sroutine medical care for kidney disease or from reference tissue as described.
Ethics oversight	We have complied with all ethical regulations related to this study. All experiments on human samples followed all relevant guidelines and regulations. The relevant oversight information is given based on tissue sources. Samples as part of the HuBMAP consortium were collected by the Kidney Translational Research Center (KTRC) under a protocol approved by the Washington University Institutional Review Board (IRB #201102312). Human reference nephrectomy specimens were obtained from the Biopsy Biobank Cohort of Indiana and KPMP under IRB #1906572234 from Indiana University.

Note that full information on the approval of the study protocol must also be provided in the manuscript.

Field-specific reporting

Please select the one below that is the best fit for your research. If you are not sure, read the appropriate sections before making your selection.

☒ Life sciences ☐ Behavioural & social sciences ☐ Ecological, evolutionary & environmental sciences

For a reference copy of the document with all sections, see nature.com/documents/nr-reporting-summary-flat.pdf

Life sciences study design

All studies must disclose on these points even when the disclosure is negative.

Sample size	Sample sizes were not predetermined by statistical methods due to the nature of this study. All available samples were used. Biopsy tissue specimens from human kidneys are a limited resource, and the strength of the study lies in in the number of individuals analyzed, technologies represented for orthogonal validation and cells analyzed. Over 47,217 nuclei were included in the multiome atlas, from which labels were derived from a larger (>280,000) cell/nuclei atlas recently published. This high number of nuclei provided the power to detect all major cell types. For CUT&RUN and whole genome bisulfite sequencing, all available samples were included.
Data exclusions	No samples or data were excluded.
Replication	Two types of replication and validation are present. First, extensive quality control data is run for the multiome, CUT&RUN, and WGBS including assessment of technical replicates. Multiple supplemental figures are devoted to relaying quality control metrics to the reader. Second, we used orthogonal molecular assays and cross validation with different technologies to strongly support our findings, which could be considered analogous to replication. All the experimental data is presented in the figures, supplementary information and source data. Replicates, when shown, are biological or technical, but clearly delineated in Supplemental table 1 and in the methods.
Randomization	The assays were performed on all specimens available to us, and the results were obtained if the assays met quality control. No randomization

	was performed because this is not a prospective study and is not indicated. Generation of data and processed files were agnostic to the disease conditions.
Blinding	The investigators were not blinded because this is not a prospective interventional study. All human specimens used in this study were deidentified, however select attributes (condition, age, sex) were available to all investigators. A majority of the analyses were not performed blind as these sample attributes were needed for accurate annotation of cell types and for the design of downstream analyses. The ST imaging performed was large scale on entire specimens and the analysis was global, independent of any sampling, and agnostic to the disease condition.

Reporting for specific materials, systems and methods

We require information from authors about some types of materials, experimental systems and methods used in many studies. Here, indicate whether each material, system or method listed is relevant to your study. If you are not sure if a list item applies to your research, read the appropriate section before selecting a response.

Materials & experimental systems

n/a	Involved in the study	
☐	☒	Antibodies
☐	☒	Eukaryotic cell lines
☒	☐	Palaeontology and archaeology
☒	☐	Animals and other organisms
☒	☐	Clinical data
☒	☐	Dual use research of concern
☐	☐	Plants

Methods

n/a	Involved in the study	
☒	☐	ChIP-seq
☒	☐	Flow cytometry
☒	☐	MRI-based neuroimaging

Antibodies

Antibodies used	Antibodies used in this study for CUT&RUN reactions: H3K27ac (Cell Signaling, 8173, lots # 6 and 8), H3K27me3 (Cell Signaling, 9733, lot #16), H3K4me1 (Cell Signaling, 5326, lot #5), H3K4me3 (Cell Signaling, 9751, lot #14), and IgG (Cell Signaling, 2729, lot #9) at a 1:50 dilution.
Validation	Orthogonal validation was provided using multiome sequencing and WGBS sequencing, showing strong alignment of epigenetic features. Additional Antibody validation information is given here:<br><br>For antibody validation/specificity/application:<br>Cell Signaling 8173, H3K27ac: from manufacturer's website, "Acetyl-Histone H3 (Lys27) (D5E4) XP® Rabbit mAb recognizes endogenous levels of histone H3 protein only when acetylated at Lys27. This antibody does not cross react with histone H3 acetylated at Lys9, 14, 18, 23, or 56. This antibody shows some cross-reactivity with acetyl-histone H2B lysine 5." Species reactivity includes Human, Mouse Rat, Monkey, Hamster, Xenopus, Zebrafish, Horse, Guinea Pig, Rabbit. Applications for this antibody from the manufacturer include western blot, immunofluorescence, flow cytometry, ChIP, and Cut&Run. Cut&Run was performed with HeLa cells using Cut&Run Assay kit #86652. In addition, the following publication used this antibody for Cut&Run Nature Communications volume 14, Article number: 4404 (2023).<br><br>Cell Signaling 9733 H3K27me3: from manufacturer's website, "Tri-Methyl-Histone H3 (Lys27) (C36B11) Rabbit mAb detects endogenous levels of histone H3 only when tri-methylated on Lys27. The antibody does not cross-react with non-methylated, mono-methylated or di-methylated Lys27. In addition, the antibody does not cross-react with mono-methylated, di-methylated or tri-methylated histone H3 at Lys4, Lys9, Lys36 or Histone H4 at Lys20." Species reactivity includes Human, Mouse, Rat, Monkey, Xenopus, Zebrafish. Applications for this antibody from the manufacturer include western blot, immunohistochemistry, immunofluorescence, flow cytometry, ChIP, and Cut&Run. Cut&Run was performed with HeLa cells using Cut&Run Assay kit #86652. In addition, the following publication used this antibody for Cut&Run Nature Protocols volume 13, pages1006–1019 (2018).<br>Cell Signaling 5326 H3K4me1: from manufacturer's website: "Mono-Methyl-Histone H3 (Lys4) (D1A9) XP® Rabbit mAb detects endogenous levels of histone H3 only when mono-methylated on Lys4. The antibody does not cross-react with non-methylated, di-methylated or tri-methylated histone H3 Lys4. In addition, the antibody does not cross-react with methylated histone H3 Lys9, Lys27, Lys36 or methylated histone H4 Lys20." Species reactivity includes Human, Mouse, Rat, Monkey, and D. melanogaster. Applications for this antibody from the manufacturer include western blot, immunofluorescence, flow cytometry, ChIP, and Cut&Run. Cut&Run was performed with HeLa cells and Cut&Run Assay Kit #86652. In addition, the following publication used this antibody for Cut&Run, Molecular Cell Volume 83, Issue 14, 20 July 2023, Pages 2398-2416.e12.<br><br>Cell Signaling 9751 H3K4me3: from manufacturer's website: "Tri-Methyl-Histone H3 (Lys4) Antibody detects endogenous levels of histone H3 when tri-methylated on Lys4. This antibody shows some cross-reactivity with histone H3 that is di-methylated on Lys4, but does not cross-react with non-methylated or monomethylated histone H3 Lys4. In addition, the antibody does not cross-react with methylated histone H3 Lys9, Lys27, Lys36 or methylated histone H4 Lys20." Species reactivity includes Human, Mouse, Rat, Monkey, D. melanogaster, S. cerevisia, Xenopus, Zebrafish. Applications for this antibody from the manufacturer include western blot, immunohistochemistry, immunofluorescence, flow cytometry, ChIP, and Cut&Run. Cut&Run was performed with HCT 116 cells and Cut&Run Assay Kit #86652. In addition, the following publication used this antibody for Cut&Run Nature Communications volume 14, Article number: 4404 (2023).

Cell Signaling 2729 IgG: from the manufacturer's website: "Normal Rabbit IgG is an unconjugated rabbit polyclonal antibody that is routinely used as a non-specific IgG control in chromatin immunoprecipitation using our SimpleChIP® Enzymatic Chromatin IP Kits #9002 and #9003." Applications include immunoprecipitation and ChIP.

Eukaryotic cell lines

Policy information about cell lines and Sex and Gender in Research

Cell line source(s)	The only cell line used was a Normal Human Proximal Tubular Kidney (NHPTK) cell. This primary cell line was generated from a human kidney (53 yo Male) and immortalized by telomerase elongation in our lab group. These cells have been extensive characterized in publications, but are not commercially available. PMID: 23383103, PMID: 28335481
Authentication	Because this cell line was generated in our lab group and extensively characterized, authentication was not indicated.
Mycoplasma contamination	All cell types were negative for mycoplasma contamination.
Commonly misidentified lines (See ICLAC register)	none

Plants

Seed stocks	*Report on the source of all seed stocks or other plant material used. If applicable, state the seed stock centre and catalogue number. If plant specimens were collected from the field, describe the collection location, date and sampling procedures.*
Novel plant genotypes	*Describe the methods by which all novel plant genotypes were produced. This includes those generated by transgenic approaches, gene editing, chemical/radiation-based mutagenesis and hybridization. For transgenic lines, describe the transformation method, the number of independent lines analyzed and the generation upon which experiments were performed. For gene-edited lines, describe the editor used, the endogenous sequence targeted for editing, the targeting guide RNA sequence (if applicable) and how the editor was applied.*
Authentication	*Describe any authentication procedures for each seed stock used or novel genotype generated. Describe any experiments used to assess the effect of a mutation and, where applicable, how potential secondary effects (e.g. second site T-DNA insertions, mosiacism, off-target gene editing) were examined.*
